# Signal peptide cleavage is essential for surface expression of a regulatory T cell surface protein, leucine rich repeat containing 32 (LRRC32)

**DOI:** 10.1186/1471-2091-12-27

**Published:** 2011-05-26

**Authors:** Derek V Chan, Ally-Khan Somani, Andrew B Young, Jessica V Massari, Jennifer Ohtola, Hideaki Sugiyama, Edina Garaczi, Denise Babineau, Kevin D Cooper, Thomas S McCormick

**Affiliations:** 1 Previous Address: Department of Dermatology, University Hospitals Case Medical Center and Case Western Reserve University, Cleveland, OH, 44106 USA; 2 Department of Dermatology and Allergology, University of Szeged, Szeged, Hungary; 3 Department of Dermatology, University of Yamanashi, Yamanashi, Japan; 4 Statistical Sciences Core, Center For Clinical Investigation, Case Western Reserve University, Cleveland, OH, 44106 USA; 5 VA Medical Center, Cleveland, OH, 44106 USA; 6 Current Address: Ohio State University Dermatology, 2012 Kenny Road, Columbus, OH, 43221, USA; 7 Current Address: Department of Dermatology, Indiana University School of Medicine, 550 N. University Blvd., Suite 3240, Indianapolis, IN, 46202, USA

## Abstract

**Background:**

Elevated numbers of regulatory T cells (T_regs_) have been implicated in certain cancers. Depletion of T_regs _has been shown to increase anti-tumor immunity. T_regs _also play a critical role in the suppression of autoimmune responses. The study of T_regs _has been hampered by a lack of adequate surface markers. Leucine Rich Repeat Containing 32 (LRRC32), also known as Glycoprotein A Repetitions Predominant (GARP), has been postulated as a novel surface marker of activated T_regs_. However, there is limited information regarding the processing of LRRC32 or the regulatory phenotype and functional activity of T_regs _expressing LRRC32.

**Results:**

Using naturally-occurring freshly isolated T_regs_, we demonstrate that low levels of LRRC32 are present intracellularly prior to activation and that freshly isolated LRRC32^+ ^T_regs _are distinct from LRRC32^- ^T_regs _with respect to the expression of surface CD62L. Using LRRC32 transfectants of HEK cells, we demonstrate that the N-terminus of LRRC32 is cleaved prior to expression of the protein at the cell surface. Furthermore, we demonstrate using a construct containing a deleted putative signal peptide region that the presence of a signal peptide region is critical to cell surface expression of LRRC32. Finally, mixed lymphocyte assays demonstrate that LRRC32^+ ^T_regs _are more potent suppressors than LRRC32^- ^T_regs_.

**Conclusions:**

A cleaved signal peptide site in LRRC32 is necessary for surface localization of native LRRC32 following activation of naturally-occurring freshly-isolated regulatory T cells. LRRC32 expression appears to alter the surface expression of activation markers of T cells such as CD62L. LRRC32 surface expression may be useful as a marker that selects for more potent T_reg _populations. In summary, understanding the processing and expression of LRRC32 may provide insight into the mechanism of action of T_regs _and the refinement of immunotherapeutic strategies aimed at targeting these cells.

## Background

Leucine Rich Repeat Containing 32 (LRRC32), also known as Glycoprotein A Repetitions Predominant (GARP), is a member of the leucine rich repeat family that exhibits evolutionary similarity to Toll-like receptors [1]. It was initially localized to chromosome *11q13-14 *but has since been further defined and mapped to the *11q13.5-14 *region [2-5]. The *Lrrc32 *gene consists of two coding exons and is expressed as two different transcripts, 4.4 and 2.8 kb in length [3]. The homologous mouse gene has been found in the *7F *chromosomal region and shares high sequence homology to human LRRC32 [2,6]. The homologous gene in grass carp (*Ctenopharyn-godonidellus*) shares 37% homology with human *Lrrc32*, and it has been found to contain transcription factor binding sites for AP1, IRF-1, IRF4, IRF-7, and NFAT, which are critical for the expression of many cytokines, suggesting a role for LRRC32 in the immune system [7].

*Lrrc32 *has been shown, via Northern blot, to be expressed in placenta, lung, kidney, heart, liver, skeletal muscle, and pancreas but not brain [3]. Furthermore, *Lrrc32 *mRNA is also highly expressed in activated T_regs _and appears to mediate FoxP3 expression, enabling T_regs _to suppress effector cell activation [8-10]. With respect to the structural aspects of LRRC32, a sequence analysis of the human 662 amino acid protein product initially suggested that it was almost entirely extracellular, with 20 leucine rich repeats in the extracellular portion of the protein followed by a hydrophobic stretch of proteins thought to be a transmembrane domain, followed by a short cytoplasmic domain consisting of 15 amino acids [3]. Sequence analysis by SignalP 3.0 revealed a putative N terminal signal peptide with a likely cleavage site after residue 17 of the pro-peptide. Surface localization of this protein in transfected cells has been confirmed [8,9,11]. N-linked glycosylation has also been reported to play a role in post-translational processing of this protein [3].

LRRC32 is expressed on the surface of expanded T_regs_, and IL-2-expanded LRRC32-positive CD25^hi ^cells are more suppressive than their IL-2-expanded LRRC32-negative counterparts [10]. Inhibition of LRRC32 expression using lentiviral or siRNA strategies in expanded T_reg _populations results in decreased suppressive capacity of the targeted cells [10]. The studies summarized above used previously-expanded T_regs_. Therefore, they do not address the suppressive capacity of freshly isolated naturally-occurring LRRC32^+ ^and LRRC32^- ^subpopulations of CD25^hi ^regulatory cells. Although addition of TGF-β to LRRC32^-^CD25^- ^cells induced FoxP3 expression, LRRC32 was not upregulated, and cells treated in this manner were unable to suppress the proliferation of T effectors, suggesting that the upregulated expression of FoxP3 was not sufficient to confer suppressive capacity on effector cells [10]. Furthermore, over-expression of FoxP3 on activated CD4^+ ^T cells did not induce expression of LRRC32 on the cell surface [10]. Finally, it has also recently been reported that LRRC32 binds latency-associated protein (LAP) and that surface expression of LAP, in turn, is upregulated on activated T_regs _in conjunction with LRRC32 upregulation [11-13]. As previously reported, T_regs _may also use cell-surface bound transforming growth factor beta (TGF-β) to directly inhibit T_eff _growth in a cell to cell contact dependent manner [14].

Naturally-occurring T_regs _are currently defined by the phenotypic expression of numerous surface markers including CD4, CD25, CD127, CD49, GITR, CTLA4, and the intracellular transcription factor FoxP3 [15-22]. Since no single marker identifies the T_reg _subset, the potential use of LRRC32 as an additional surface marker for potent T_regs _is of interest. We surmised that LRRC32 surface expression on T_regs _might have utility for the selection of T_regs _for functional studies as well as differentiation and activation studies. Although previous studies have looked at the functional suppressive capacity of T_regs _that were expanded with cytokines such as IL-2, we chose to study the suppressive capacity of naturally-occurring freshly isolated activated T_regs _in the absence of long-term culture or repeated rounds of stimulation.

A previous study utilized a signal peptide deletion construct to show that naive T cells transfected with an LRRC32 signal peptide deletion construct lacked protein upregulation of CD25, CD62L, and FoxP3 compared to transfection with wildtype LRRC32 [8]. This study utilized GFP-tagged signal peptide deletion constructs that were transfected into *HEK293 *cells to study the contribution of signal peptide to surface expression of LRRC32 [8]. Surface expression was only evaluated by phase contrast and DAPI confocal microscopy of single cells. These images suggested that some of the LRRC32 signal traffic to the cell surface, in contrast with our prediction. Because experiments by Wang *et al*. were unclear and did not conclusively show that deletion of the signal peptide region affects cell surface expression of conformationally intact native LRRC32 using antibodies capable of recognizing the extracellular domain of LRRC32, we decided to characterize the functional and phenotypic properties of T_regs _expressing LRRC32 by immunuohistochemistry.

We show that LRRC32 is a marker for a more potent subset of freshly isolated activated T_regs_. We further characterize T_reg _subsets with respect to the expression of other T_reg _markers in the context of LRRC32 expression. We examine the intracellular processing of LRRC32 and conclusively demonstrate in multiple cells that the N-terminal portion of LRRC32 is cleaved prior to expression of the protein on the cell surface and that cleavage of this signal peptide is necessary for translocation of the mature protein to the cell surface. Furthermore, we directly confirm, using antibodies specific for native LRRC32, that the signal peptide region of LRRC32 is critical for its surface expression. We also demonstrate low levels of intracellular LRRC32 prior to activation via the T cell receptor (TCR) and CD28, suggesting that low levels of LRRC32 are sequestered intracellularly and that T cell activation is necessary for the synthesis and surface expression of LRRC32. Expression of LRRC32 may enhance T_reg _function. Therefore, refinement of immunotherapeutic strategies aimed at targeting LRRC32 may improve strategies for T_reg _isolation and yield more potent T_regs_.

## Methods

### Isolation of CD4^+ ^cells

Peripheral blood was donated by healthy human volunteers coordinated by the Skin Diseases Research Center at University Hospitals Case Medical Center. Signed informed consents were obtained from volunteers prior to their participation in the study. The study protocol was approved by the Institutional Review Board of University Hospitals Case Medical Center. Peripheral blood mononuclear cells (PBMCs) were prepared by Histopaque-1077 (Sigma-Aldrich) density gradient separation in accordance with manufacturer's protocols. Negatively selected CD4^+ ^T cells were purified using magnetic bead technology, per manufacturer's instructions (Miltenyi Biotec).

### Generation of Constructs

Plasmid DNA encoding the cDNA of full length human LRRC32 protein (TrueClone pCMV6-XL6 Human Full-Length cDNA Clones, OriGene) was used to transform competent One Shot Top 10 *E. coli *(Invitrogen), according to the manufacturer's instructions. Selection of positive clones was performed on kanamycin (Invitrogen) LB agar plates. Plasmids were recovered and purified using a Qiaquick MaxiPrep purification kit (Qiagen). The following primers encoding regions flanking the entire *Lrrc32 *sequence were used to amplify the *Lrrc32 *cDNA sequence via PCR and insert a TOPO cloning site for pENTR/D-TOPO (Invitrogen): pCMV6-LRRC32 reverse (TTAG GCTTTATACTGTTGGTTAAACTT), pCMV6-LRRC32 reverse readthrough (GGCTTTATA CTGTTGGTTAAACTTCTG), and pCMV6-LRRC32 forward (CACCATGAGACCCCAGA TCCTGCT).

The following 1x PCR buffer conditions were used: AccuPrime Pfx 1x reaction buffer (Invitrogen), 10 mM of forward primer, 10 mM of reverse or reverse readthrough primer, 50 ng of template DNA, and 1 unit of AccuPrime Pfx DNA (Invitrogen). PCR conditions were as follow: initial denaturation at 95 C for 2 minutes followed by 36 cyles of denaturation, annealing, and extension at 95 C, 55 C, and 68 C for 15 s, 30, and 2 minutes, respectively, followed by final extension at 68 C for 30 minutes.

PCR products containing the *Lrrc32 *sequence derived from reactions utilizing either the forward and reverse primers or forward and reverse readthrough primers were then inserted into pENTR/D-TOPO, per the manufacturer's instructions to generate two different respective constructs: pENTR/D-TOPO/C-terminus LRRC32 (containing a stop codon at the end of the *LRR32 *sequence) and pENTR/D-TOPO/N-terminus LRRC32 (lacking a stop codon at the end of the *LRR32 *sequence). One Shot Top 10 *E. coli *were transformed as described above, and positive clones were selected using LB Agar plus kanamycin plates. Plasmid DNA was isolated using a Qiaquick MiniPrep (Qiagen) kit, and M13 forward and reverse primers were subsequently used to facilitate sequencing of the inserted PCR products within each of the screened and purified plasmids via the Sequencing Core at Case Western Reserve University. The reported sequence was aligned with the reported cDNA sequence of LRRC32 obtained from OriGene using the Vector NTI (Invitrogen) software system, and sequence alignments were then confirmed prior to further utilization of the LRRC32 sequence-verified plasmids.

LRRC32 sequence-verified pENTR/D-TOPO/N-terminus LRRC32 or pENTR D-TOPO/C-terminus LRRC32 plasmids were then used in a Gateway (Invitrogen) cloning strategy utilizing either the Vivid Colors pcDNA6.2/N-EmGFP or pcDNA6.2/C-EmGFP (Invitrogen) as destination vectors, per manufacturer's instructions to generate two products, pcDNA6.2 N-terminus LRRC32/C-EmGFP and pcDNA6.2 C-terminus LRRC32/N-EmGFP, encoding "C-GFP/LRRC32" and "N-GFP/LRRC32" respect-ively. Products were used to transform One Shot Top 10 *E. coli*., and selection of positive clones was performed on LB Agar plus ampicillan plates. Purified plasmids derived from expanded clones were screened again using restriction enzyme analysis to confirm expected restriction sites in the sequence.

To generate an *Lrrc32 *signal peptide deletion construct, we utilized the newly created pcDNA6.2 C-terminus LRRC32/N-EmGFP vector as a template for PCR amplification. The pCMV6-LRRC32 reverse readthrough primer, described above, as well as a newly created pCMV6-LRRC32ΔSP primer (CACCATGGCACAACA CCAAGACAAAGT), designed to anneal optimally to bases just distal to the presumed signal peptide cleavage site of LRRC32, were utilized to generate a PCR product containing the *Lrrc32 *sequence without a putative signal peptide sequence. The PCR product was then inserted into pENTR/D-TOPO, and an identical strategy as that described above was utilized to create a pcDNA6.2 N-terminus LRRC32ΔSP/C-EmGFP product coding for a LRRC32 signal peptide deletion construct ("C-GFP/LRRC32ΔSP") tagged at the C-terminus end with GFP. The primer "C-EmGFP TOPO Internal Rev" (TGAACTTCAGGGTCAGCTTGCCGTA) was utilized to confirm that the GFP and LRRC32 (minus the signal peptide) coding regions were in frame.

### Realtime PCR Analysis

RNA was extracted using the RNeasy kit (Qiagen). 100 ng of total RNA was then processed using the SuperScript III First-Strand Synthesis System (Invitrogen) and random hexamers as primers and to generate cDNA, per manufacturer's protocol. The following gene expression assays were subsequently used for RT-PCR analysis: FoxP3 (HS00203958_ml, Applied Biosystems), LRRC32 (HS00194136_ml, Applied Biosystems), 18S (HS99999901_sl, Applied Biosystems), and GFP (Applied Biosystems). RT-PCR analysis was performed in accordance with the manufacturer's suggested protocol (Applied Biosystems) with an Applied Biosystems 7500 Realtime PCR system, and StepOne 2.0 analysis software was used to analyze the data.

### Transfection of and Establishment of Stable Cell Lines

*HEK293 *cells (ATCC, Manassas, VA) were transfected with plasmid containing either pENTR D-TOPO/C-terminus LRRC32 ("C-GFP/LRRC32") or N-terminus LRRC32 ("N-GFP/LRRC32"), utilizing a 3:1 ratio of FuGene 6 (microliters, Roche) to plasmid (micrograms) according to the manufacturer's protocol. Stable transfectants were selected using blastocidin (Invitrogen) according to the manufacturer's protocol, and individual cells were sorted based upon their GFP expression using a FACS Aria cell sorting system (Becton Dickinson) into 96 well plates. Stable clones expressing N-GFP/LRRC32, C-GFP/LRRC32, or C-GFP/LRRC32ΔSP were thus derived from sorted single cells with the highest GFP expression.

### Biotinylation of cell surface proteins, Immunoprecipitation and Western Blotting

Sulfo-NHS-LC-Biotin (#21327, Pierce) was utilized to label cell surface proteins, in accordance with the manufacturer's protocol. Biotinylated cells were lysed in Glo Lysis Buffer (#E266A, Promega) per manufacturer protocols. GFP-tagged proteins derived from the cell lysates were then immunoprecipitated with mouse anti-GFP (#A11120, Invitrogen) using an ExactaCruz E system, per manufacturer's instructions (#sc-45042, Santa Cruz Biotechnology). Immunoprecipitated proteins were electrophoresed on precast NuPAGE gels (Invitrogen) with their corresponding premade MOPS SDS buffers (Invitrogen). Proteins were transferred to polyvinylidene difluoride (PVDF) membranes (#LC2002, Invitrogen), per manufacturer's protocol, for subsequent probing using anti-GFP antibody (#A11121, Invitrogen; #33-2600, Zymed) or isotype control antibody (Invitrogen). A SuperSignal PICO (Pierce) kit containing HRP-coupled rabbit anti-mouse antibody was used for detection of GFP-tagged proteins. HRP-coupled goat polyclonal to mouse IgG (#ab6789, Abcam) was also used to recognize bound murine antibody. Rabbit polyclonal antibodies to Foxp3 (#ab10563, Abcam) were used to probe for human FoxP3 on membranes, and HRP-coupled goat polyclonal antibodies to rabbit IgG were used to recognize bound rabbit antibodies. For detection of biotinylated proteins, streptavidin-HRP (#21126, Pierce) was utilized.

For the assessment of cellular LRRC32 expression in the context of the signal peptide deletion constructs, an identical protocol as above was used except that cells were lysed using the M-PER Mammalian Protein Extraction Reagent (#78503, Pierce). Plato-1(ALX-804-867-C100, Axxora) was used to detect LRRC32, and the Pierce Fast Western Blot Kit (#35050, Pierce) was used to visualize immunoblots.

### Flow Cytometry Analysis of CD4^+ ^Cells

**Figure 1:**
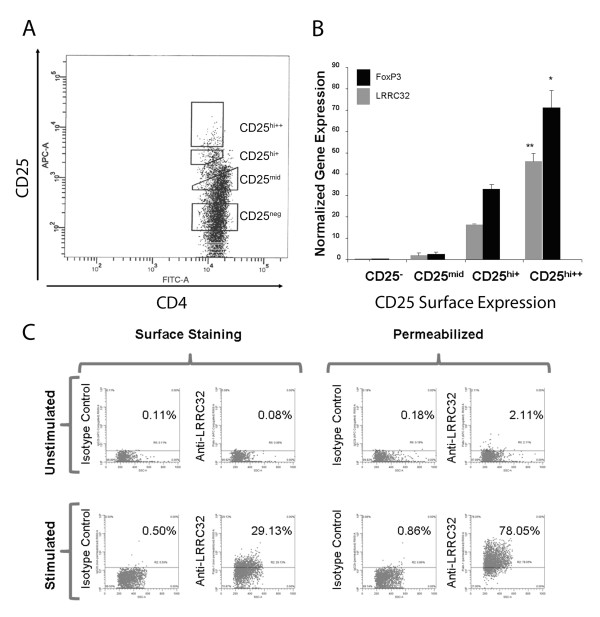
***Lrrc32 *mRNA is preferentially expressed in naturally-occurring freshly-isolated non-expanded human T_regs _but is not observed on the surface of these cells**. a) T cell sorting gates based upon CD25 surface expression. b) *Lrrc32 *mRNA expression comports with *Foxp3 *mRNA expression and CD25 surface expression, and *Lrrc32 *mRNA is preferentially expressed in T_regs_, compared to T_effs_. Relative expressions of *18SrRNA*-normalized *Foxp3 *and *Lrrc32 *genes were determined using real-time PCR. Data summarize four independent experiments. Results are expressed as the mean ± SEM. *p = 0.01 compared to CD25^mid ^cells, **p = 0.005 compared to CD25^mid ^cells. c) Flow cytometric analysis of freshly-isolated activated CD4^+ ^human T cells shows that the CD25^hi ^subgroup (composed of the CD25hi+ and CD25hi++ subgroups denoted in figure 1b) demarcating T_regs _expresses LRRC32 on the cell surface (top right panel) but that the CD25^- ^subgroup demarcating T_effs _expresses negligible amounts of LRRC32 on the cell surface (bottom right panel). Analysis strategy is indicated via the arrows. d) Flow cytometric analysis of sorted resting CD4^+ ^human T_regs _shows that they lack surface expression of LRRC32 protein (top left panel). However, after permeabilization of the cell membrane, a low expression of LRRC32 protein can be observed intracellularly (top right panel). After activation of the sorted T_regs _with a T_reg _expansion kit, LRRC32 can be seen on the surface of these activated T_regs _(bottom left panel), and evidence of intracellular expression of LRRC32 can also be observed after permeabilization of the cell membrane (bottom right panel).

CD4^+ ^cells were placed in culture media (RPMI, 10% FBS, penicillin, streptomycin, L-glutamine, and β-mercaptoethanol) and rested overnight in 96-well plates or stimulated in anti-CD3-coated plates supplemented with soluble murine anti-human CD28 (1 microgram/ml, Becton Dickenson) [23]. Cells were subsequently stained using antibodies to CD25 (clone 2A3, Becton Dickenson), LRRC32 (Axxora), FoxP3 (eBioscience), and a panel of antibodies specific for CD69 (Becton Dickinson), CD62L (Becton Dickinson), GITR (R & D Biosystems), CTLA4 (Becton Dickinson), HLA-DR (Becton Dickinson), and CD45RO (Becton Dickinson), per manufacturer's protocol for staining cells for flow cytometry (eBioscience). Cells were fixed and then permeabilized using a fixation permeabilization kit (eBioscience) after staining surface antigens in order to study the intracellular expression of certain proteins (LRRC32, FoxP3). For assessment of intracellular expression of LRRC32, permeabilized cells were incubated with 2.5 micrograms/ml of IgG2b control isotype antibody (Invitrogen) for 30 minutes prior to incubation with labeled anti-LRRC32 antibody to decrease non-specific binding. Isotype controls were performed for compensation. Cells were analyzed on a Becton Dickenson LSR II flow cytometer. In all instances, with the exception of the mRNA studies, CD25^hi ^cells represented the top 5% of CD25^+ ^cells. In the case of the mRNA studies in which CD25^hi ^and ^mid ^populations were identified, CD25^hi++ ^cells represented the top 1.5% of CD25^+ ^cells, and CD25^hi+ ^cells represented the next highest 2-3% of CD25^+ ^cells as depicted in Figure 1a.

### Expansion and stimulation of T_regs_

CD4^+ ^cells were isolated as described above. Subsequently, the cells were stained with anti-CD25 (Becton Dickenson). The top 5% of CD25^hi ^cells were sorted, and cells were expanded and stimulated for 2 weeks using beads coated with anti-CD3 and anti-CD28 according to manufacturer directions (Dynabeads Human T_reg _Expander, Catalog #111.29, Invitrogen) prior to characterization. Unstimulated sorted CD25^hi ^cells derived from isolated CD4^+ ^cells from the same patient were used as a control for unsimulated T_regs_.

### Proliferation Assay

Negatively selected CD4^+ ^cells were stained with anti-CD25, and the top 5% of CD25 cells (CD25^hi^) were sorted using a Becton Dickenson FACS Aria cell sorter. Isolated cells were stimulated overnight on anti-CD3-coated plates (Becton Dickinson) supplemented with soluble murine anti-human CD28 (Pharmingen) as described above. CD25^- ^effector T cells were maintained in culture media. Stimulated CD25^hi ^cells were subsequently stained with antibody specific for LRRC32 (Axxora), and LRRC32^+ ^and LRRC32^- ^subpopulations were further sorted. LRRC32^+ ^positive and LRRC32^- ^T_reg _subpopulations were then co-cultured with CD25^- ^effector T cells plus irradiated allogeneic antigen presenting cells (APCs) at various T_reg_:T_eff _ratios ranging from 1:1 to 1:16 in a 96 well round bottomed tissue culture plate (Costar) as previously described [23]. Each well contained 20,000 T_effs _and 50,000 APCs which had been previously irradiated at 3,000 Rad. Mixed lymphocytes were cultured for 6 days, and cells were pulsed with 1 μCi/well [^3^H]thymidine (NET-027A, Perkin-Elmer) for the last 16 h. Proliferation was measured using a TopCount scintillation counter (Perkin-Elmer). Maximum proliferation was calculated by measuring the proliferation of cells in wells lacking T_regs _and containing only T_effs _and APCs. Background proliferation was ascertained via measurement of proliferation of T_effs _only in the absence of APCs.

### Statistical Analysis

Statistical analysis was performed using Student's t test (surface phenotype analysis) or a three way ANOVA (mixed lymphocyte response assays) as indicated in the figure legends. A value of *p *= 0.05 was considered significant, unless otherwise indicated.

For the ANOVA, the overall *p *value reported is reflective of the difference between the average proliferation of T_regs _using LRRC32 as an independent variable while controlling for the individual assays as well as the titration. The *R*^*2 *^value represents the amount of variability in the ANOVA analysis that is accounted for by the presence or absence of LRRC32 on the surface of a T_reg_, the individual assays, as well as the titration.

## Results

### Lrrc32 Is Preferentially Expressed in T_regs_

A preliminary microarray analysis demonstrated upregulated *Lrrc32 *gene expression in T_regs _[9]. To confirm these findings, we performed RT PCR analysis examining LRRC32 mRNA expression in sorted subpopulations of CD25-expressing cells as depicted (Figure 1a) representing 1.5% of the CD4^+ ^cell population with the highest expression of CD25 (CD25^hi++^), 2-3% of the CD4^+ ^cell population with high expression of CD25 (CD25^hi+^), 17-20% of the CD4^+ ^cell population with reduced expression of CD25 (CD25^mid^), or no expression of CD25 (CD25^-^). These analyses revealed that *Lrrc32 *expression increased with surface CD25 expression in a manner similar to *Foxp3 *expression (Figure 1b), confirming that *Lrrc32 *is preferentially expressed in T_regs_. To confirm that LRRC32 is specifically upregulated in activated T_regs_, we compared the surface expression of LRRC32 in the CD25^hi ^population and the CD25^- ^T_eff _population of CD4^+ ^cells stimulated overnight with plate bound anti-CD3 and soluble anti-CD28 and found that LRRC32 is preferentially expressed on the surface of activated T_regs _but not activated T_effs _(Figure 1c), in accordance with a previous study [10]. Furthermore, in accordance with this previous study, we also confirmed that surface LRRC32 is present on sorted CD4^+^CD25^hi ^T_regs _representing 5% of the CD4^+ ^cell population with the highest expression of CD25 (i.e. including both the CD25^hi++ ^and CD25^hi+ ^subgroups described above, Figure 1d, surface expression, stimulated, bottom left panel set) that had been activated for 2 weeks with beads coated with anti-CD28 and anti-CD3 but not on unstimulated sorted CD4^+^CD25^hi ^T_regs _(Figure 1d, surface expression, unstimulated, top left panel set) [10]. A low intracellular expression of LRRC32 in resting sorted CD4^+^CD25^hi ^T_regs _was noted (Figure 1d, permeabilized expression, unstimulated, top right panel set). This suggests that low levels of LRRC32 may be sequestered in T_regs _or might require additional processing prior to surface expression. Increased expression of LRRC32 could be seen in activated CD4^+^CD25^hi ^sorted Tregs that had been permeabilized (Figure 1d, permeabilized expression, stimulated, bottom right panel set) and was higher than that seen on the surface only (Figure 1d, surface expression, stimulated, bottom left panel set), as would be expected for a surface protein that is initially produced intracellularly upon cell activation before trafficking to the cell surface.

### Lrrc32 Is Cleaved After Processing

**Figure 2:**
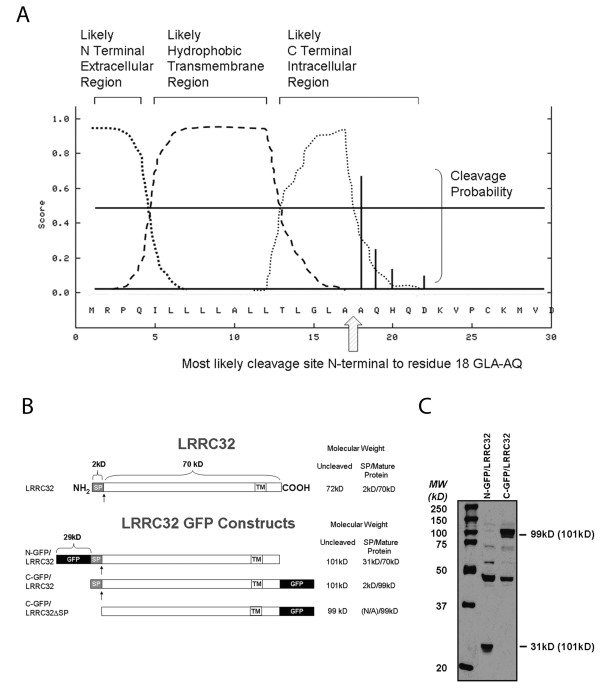
**LRRC32 contains an N-terminal signal peptide and a transmembrane region**. a) Analysis of the amino acid sequence of LRRC32 by SignalP 3.0 software predicted a putative N-terminal cleavage site between alanines 17 and 18. Alternative potential cleavage sites are predicted and indicated as vertical solid lines on amino acids 19, 20, and 22. b) To address the actual cleavage site, N-terminal and C-terminal GFP-tagged constructs were designed as depicted and generated to facilitate further analysis of surface expression and cleavage. SP = signal peptide. TM = transmembrane region. GFP = green fluorescent protein. Arrow = putative cleavage site. c) Anti-GFP immunoblot analysis of total lysates from C-and N- terminal expressing clones revealed 99 kD (29 kD GFP + 70 kD LRRC32) and 31 kD (29 kD GFP + 2 kD LRRC32 signal peptide) fusion proteins respectively. Expected sizes of uncleaved fusion proteins, based upon predicted protein sequences, are shown in parentheses. The difference in protein size between the C- and N-terminal fusion proteins confirms a cleavage site in the N-terminus of the protein between alanines 17 and 18.

Sequence analysis of murine LRRC32 predicted that LRRC32 would be expressed as a transmembrane protein [6]. We analyzed the sequence of human LRRC32 using the transmembrane domain prediction server at Stockholm Bioinformatics Center with a dense alignment method and confirmed the existence of a putative 19 amino acid transmembrane domain incorporating residues 628 through 646 (data not shown), in agreement with prior reports that this protein was expressed at the cell surface [10,11]. Using the SignalP 3.0 server, we also identified a putative 17 amino acid signal peptide sequence and cleavage site between residues 17 and 18 (Figure 2a), consistent with the requirement for a signal peptide to be present in a protein destined for expression at the cell surface [24]. To further understand protein processing and surface expression of human LRRC32, we generated LRRC32 constructs incorporating green fluorescent protein (GFP) at either the N- or C- terminus, thereby allowing us to examine the tagged signal peptide or the mature protein, respectively (Figure 2b). Using anti-GFP antibodies, we examined total lysates from HEK-transfected C- and N- terminus tagged LRRC32-expressing clones (Figure 2c). Here, we show that 99kD (29 kD GFP+70 kD LRRC32) and 31 kD (29 kD GFP+2 kD LRRC32) fusion proteins, respectively, are generated in HEK transfectants overexpressing LRRC32, consistent with a cleaved N-terminus signal peptide generated prior to surface expression of LRRC32.

### Lrrc32 on Transfected HEK293 Cells Is Expressed at the Cell Surface Regardless of Stimulation

**Figure 3:**
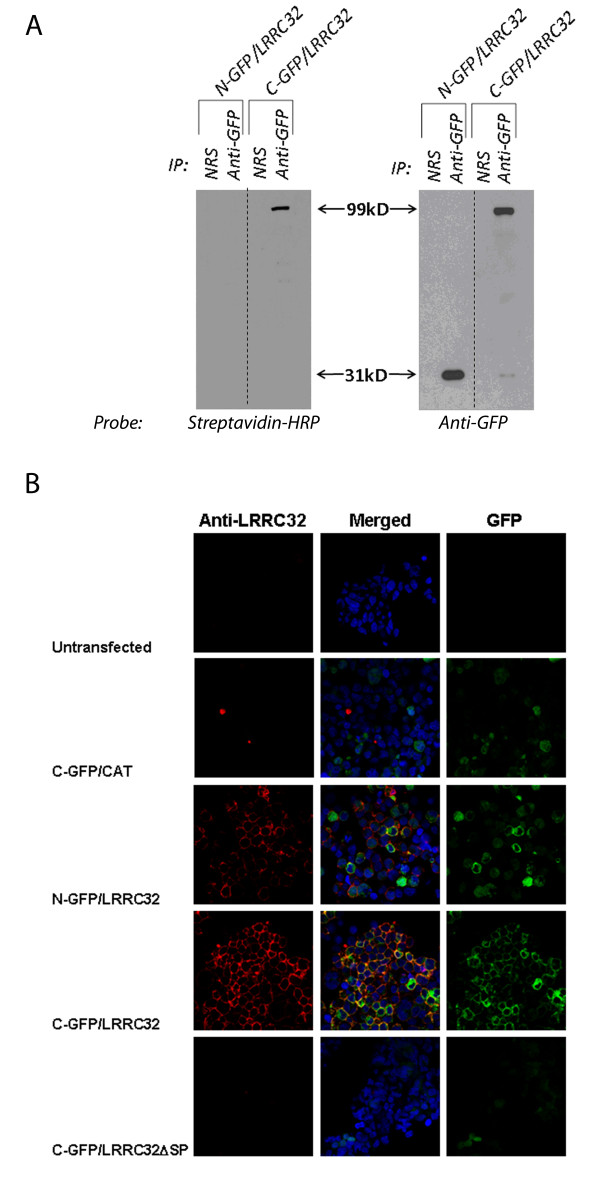
**LRRC32 is a cell surface protein**. a) C-and N- terminus GFP-tagged LRRC32 expressing *HEK293 *cell clones were surface biotinylated, and cell lysates were immunoprecipitated using antibody specific for GFP or using normal rabbit serum (NRS) as a control (left and right panels). Protein lysates were then electrophoresed, transferred to membrane PDVF, and probed for the presence of biotinylation using streptavidin-HRP (left panel only). Blots were also probed with anti-GFP (right panel only). b) Confocal analysis of untransfected (top row) HEK293 cells, C-GFP/CAT-transfected HEK293 cells (second row), N-GFP/LRRC32-transfected HEK293 cells (third row), C-GFP/LRRC32-transfected HEK293 cells (fourth row), and C-GFP/LRRC32ΔSP-transfected HEK293 cells (last row); Green = GFP, Red = anti-LRRC32 antibody, Blue = nuclear counterstain. The left column shows anti-LRRC32 only. The right column shows GFP only. The middle column shows the merged composite confocal picture (anti-LRRC32 + GFP) with the nuclear counterstain.

Although T_regs _appear to require activation prior to expressing LRRC32 on their surface, the same is not true for transfected cell lines such as *HEK293*, and the use of the *HEK293 *cell line allowed us to easily study factors affecting the surface expression of LRRC32 in a system that would not require constant T cell isolation and activation [10,11,25]. We used C- and N-terminal GFP-tagged LRRC32 (C-GFP/LRRC32 and N-GFP/LRRC32, respectively) expressing *HEK293 *cell clones for surface biotinylation and analysis. Following surface biotinylation, cell lysates were prepared and anti-GFP antibodies were used to immunoprecipitate the fusion proteins. Precipitated protein was transferred to a PVDF membrane. The membrane was then probed for biotin using streptavidin-HRP (Figure 3a, left panel) or for GFP using an anti-GFP antibody (Figure 3a, right panel). Our results demonstrate biotinylated surface protein only in C-GFP/LRRC32-expressing clones (Figure 3a, left panel, rightmost lane), consistent with both surface membrane expression of LRRC32 and intracellular N-terminal processing (removal of the GFP-N-terminal signal peptide) prior to membrane localization. Probing the membrane with anti-GFP (Figure 3a, right panel) demonstrates the surface and intracellular portions of LRRC32 after cleavage.

Confocal studies with C-terminal GFP-tagged LRRC32 (C-GFP/LRRC32)-transfected *HEK293 *cells confirm that GFP expression is found on the cell surface without stimulation, in accordance with predictions that LRRC32 would be found on the cell surface (Figure 3b, fourth row, right column). In contrast, N-terminal GFP-tagged LRRC32 (N-GFP/LRRC32)-transfected HEK293 cells exhibit GFP expression diffusely within cells, and GFP expression is not concentrated at cell surfaces, presumably since the GFP-tagged cleaved signal peptide of N-GFP/LRRC32 remains intracellularly after cleavage, prior to the translocation of the mature LRRC32 protein to the cell surface (Figure 3b, third row, right column). Antibody staining of surface LRRC32 confirmed that mature C-GFP/LRRC32 and N-GFP/LRRC32 traffic to the cell surface (Figure 3b, third and fourth rows, left column). Our untransfected control cell line did not express GFP or LRRC32 staining, as expected (Figure 3b, top row). Furthermore, our C-terminal GFP-tagged chloramphenicol acetyltransferase (C-GFP/CAT) control cell line did not express LRRC32 staining, as expected (Figure 3b, second row).

### A putative signal peptide region corresponding to the first 17 amino acids of Lrrc32 is required for surface protein expression of Lrrc32

**Figure 4:**
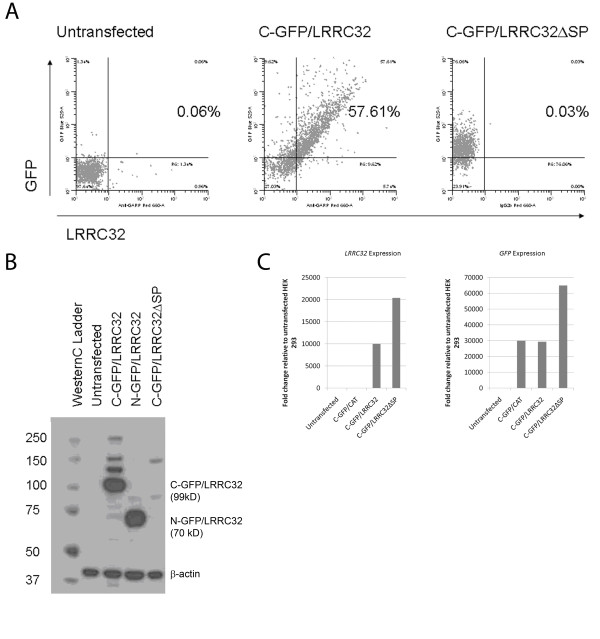
**A 17 AA signal peptide is required for the cell surface expression of LRRC32**. a) Untransfected *HEK293 *cells or cells transfected with either C-terminus GFP-tagged LRRC32 or C-terminus GFP-tagged LRRC32 with a deleted signal peptide region were analyzed by flow cytometry for surface expression of LRRC32 or GFP expression. b) Anti-LRRC32 immunoblot analysis of total lysates from C-and N- terminus GFP-tagged LRRC32 expressing clones revealed intact LRRC32 expression at 99 kD (fusion protein: 29 kD GFP + 70 kD LRRC32) and 70 kD, respectively. However, immunoblot analysis of total lysates from C- terminus GFP-tagged LRRC32 expressing clones lacking an intact signal peptide did not detect the presence of LRRC32 (rightmost lane). c) RT-PCR analysis of *HEK293 *cell lysates utilizing untransfected, C-CAT, C-terminus GFP-tagged LRRC32, or C-terminus GFP-tagged LRRC32 lacking an intact signal peptide was performed using primers for *Lrrc32 *(left panel) or *GFP *(right panel).

Since signal peptides are generally necessary to direct surface expression of proteins, we next decided to see whether deletion of the putative signal peptide region would inhibit surface expression of LRRC32 [26-28]. *HEK293 *cells transfected with a C-terminus GFP-tagged LRRC32 construct lacking the signal peptide (C-GFP/LRRC32ΔSP) did not express surface LRRC32 by confocal microscopy (Figure 3b, bottom row). Furthermore, they did not express surface LRRC32 by flow cytometry but were GFP positive, compared to *HEK293 *cells transfected with the full length C-terminus GFP-tagged LRRC32 construct, expressing both GFP and surface LRRC32 (Figure 4a). Control untransfected cells did not express GFP or surface LRRC32, as expected (Figure 4a, leftmost panel).

Given the absence of surface expression of LRRC32 in cells transfected with C-GFP/LRRC32ΔSP, we concluded that the signal peptide portion of LRRC32 was critical for surface expression of LRRC32. It was unclear, however, whether the absence of surface expression of LRRC32 was due to intracellular sequestration of LRRC32. We therefore conducted a western blot analysis of lysates from *HEK293 *cells transfected with our various constructs using an anti-LRRC32 antibody. Our results showed that while LRRC32 can be detected in cell lysates from cells transfected with full-length LRRC32 constructs (C-GFP/LRRC32 and N-GFP/LRRC32 at 99 and 70 kD, respectively), LRRC32 could not be detected in cell lysates of *HEK293 *cells transfected with the C-GFP/LRRC32ΔSP, suggesting that LRRC32 is either produced at levels that were undetectable or is rapidly broken down following translation (Figure 4b).

To confirm that *Lrrc32 *was being transcribed, we utilized RT-PCR to analyze mRNA from lysates of *HEK293 *cells transfected with our various constructs. Our analysis showed that *Lrrc32 *mRNA was detected in the C-GFP/LRRC32 as well as the C-GFP/LRRC32ΔSP-transfected *HEK293 *cell lysates (Figure 4c, left panel). In contrast, untransfected *HEK293 *cells as well as the control C-terminus tagged chloramphenicol acetyltransferase (C-CAT)-transfected *HEK293 *cell lysates had undetectable levels of *Lrrc32 *mRNA. Furthermore, as expected, GFP expression was observed in all of the *HEK293 *transfected cells examined (Figure 4c, right panel). As we utilized stable clones derived from sorted single cells with the highest GFP expression, as described above, the different efficiencies of C-GFP/LRRC32 vs. C-GFP/LRRC32ΔSP may be due to differential stability/integration of the plasmids in the selected clones. Furthermore, it is possible that the signal peptide mutant may be less stable, and as a result, cells transfected with GFP/LRRC32ΔSP may compensate by producing more mRNA to produce more protein.

### Characterization of CD62L expression and functional status of Lrrc32^+ ^and Lrrc32^- ^naturally-occurring freshly isolated human T_regs_

**Figure 5:**
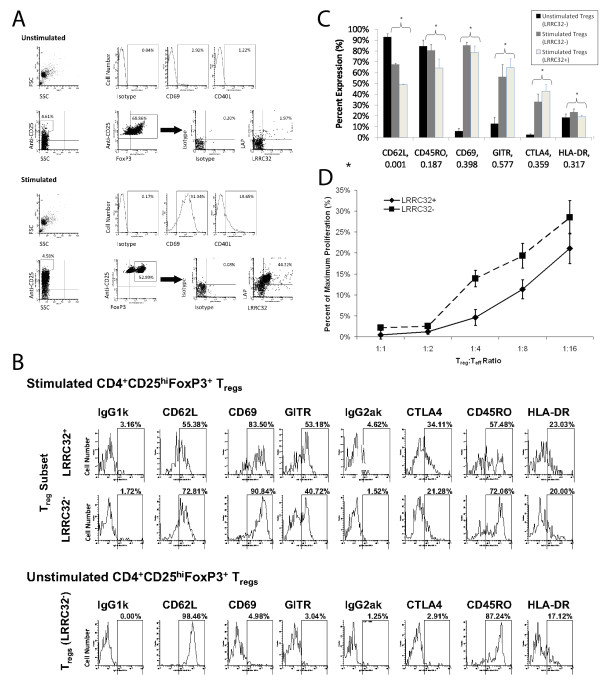
**LRRC32^+ ^CD4^+^CD25^hi^FoxP3 T_regs _appear to be more potent suppressors than LRRC32^- ^CD4^+^CD25^hi^FoxP3 and exhibit decreased CD62L upon activation**. a) Expression of LRRC32 and LAP in CD4+ T cells rested overnight (top panel) or stimulated with plate bound anti-CD3 and soluble anti-CD28 (bottom panel). T_regs _were selected from the top 5% CD25-expressing and FoxP3^+ ^populations, as previously described. Confirmation of activation by expression of the surface markers CD40L and CD69 are also shown (top of each panel). b) The expression patterns of various T_reg _and activation surface markers (CD62L, CD69, GITR, CTLA4, CD45RO, and HLA-DR) in FoxP3^+ ^and LRRC32^+^-gated populations of CD25^hi ^cells were studied using flow cytometry. Stimulated CD4^+^FoxP3^+^CD25^hi ^T_regs _(top panel) & unstimulated CD4^+^FoxP3^+^CD25^hi^T_regs _(bottom panel). c) Composite summary of phenotypic analysis of unstimulated LRRC32^-^CD4^+^CD25^hi^FoxP3^+ ^T_regs _and stimulated LRRC32^+ ^and LRRC32^- ^CD4^+^CD25^hi^FoxP3^+ ^T_regs_. Black bars = unstimulated LRRC32^- ^T_regs_. Dark grey bars = stimulated LRRC32^- ^T_regs_. Light grey bars = stimulated LRRC32^+ ^T_regs_. Data are expressed as the mean ± SEM from 3 individuals. Heteroscedastic variances and an independent t-test comparing stimulated LRRC32^+ ^and LRRC32^- ^subsets were used for calculations of the *p *values which are reported along the x-axis, below each surface marker (*). d) CD25^hi ^cells were sorted and activated overnight using anti-CD3-coated plates and soluble anti-CD28 (1 microgram/ml). Cells were then resorted based upon LRRC32 expression. The suppressive capacities of these LRRC32^+ ^and LRRC32^- ^T_regs _were subsequently tested in a mixed lymphocyte reaction utilizing syngeneic effectors (T_eff_, 20,000/well) and allogenic antigen presenting cells (50,000/well). T_reg_:T_eff _ratios are depicted above. Data summarize 3 independent experiments. Results are expressed as the mean ± SEM. *p *= 0.0001 and *R*^*2 *^= 0.7244. Absolute proliferation values for the 3 experiments were as follow: T_effs _alone: average of 31094 cpm to average of 47483 cpm (at least 6 replicates per assay), background: average of 24 cpm to 35 cpm (at least 6 replicates per assay); T_reg_:T_eff _ratio of 1:1: 89 cpm to 346 cpm. When titrating T_regs _vs. T_effs_, 3 replicates were performed at each titration for the LRRC32^+ ^and LRRC32^- ^T_regs _except for in one assay set in which there was limited number of LRRC32^+ ^T_regs_. In this case, only one replicate was performed at the 1:1 and 1:2 titrations, and two replicates were performed for the other titrations (0:1, 1:4, 1:8, and 1:16). We performed 3 replicates for each titration utilizing the LRRC32^- ^T_regs_.

Using polyclonal activation via the TCR in combination with anti-CD28 co-stimulation, we confirmed that LRRC32 is expressed on the surface of naturally-occurring freshly-isolated activated T_regs _compared to unstimulated T_regs _(24.02% ± 1.73% (n = 6) vs. 2.30% ± 1.05% (n = 6), data not shown), respectively. We subsequently confirmed that surface LAP expression is also observed in this cell population following activation with plate-bound anti-CD3 and soluble anti-CD28, in agreement with a recently published report (Figure 5a) [11].

To date, no single surface marker is sufficient for identifying naturally-occurring T_regs_. In order to address the expression of LRRC32 in the context of previously described surface markers, we analyzed LRRC32^+ ^and LRRC32^- ^subsets of CD4^+^CD25^hi^FoxP3^+^LRRC32^+ ^T_regs_, as well as unactivated T_regs_, with respect to the surface co-expression of CD62L, CD45RO, CD69, GITR, CTLA4, and HLA-DR (Figures 5b and 5c). Stimulated T_regs _demonstrated expected increases in the surface expression of GITR, CD69, and CTLA4. Of interest, however, stimulated LRRC32^+ ^T_regs _exhibited less CD62L and CD45RO than resting and stimulated LRRC32^- ^T_regs_, suggesting that LRRC32^+ ^T_regs _may represent a subset of activated or differentiated T_regs_.

To address whether or not functional differences in LRRC32^+ ^and LRRC32^- ^subsets of naturally-occurring freshly isolated T_regs _exist, we examined the suppressive capacity of LRRC32^+ ^and LRRC32^- ^T_regs_. Given previous reports that transfection of *Lrrc32*-bearing constructs into "pre-regulatory T cells" could induce them to upregulate FoxP3 expression, we hypothesized that the LRRC32^+ ^subset of naturally-occurring freshly-isolated CD4^+^CD25^hi ^T_regs _would be more suppressive than the LRRC32^- ^subset of CD4^+^CD25^hi ^T_regs _[8]. As described above, our data demonstrates that LRRC32 expression comports with FoxP3^+ ^expression. Therefore, we first sorted CD25^hi ^CD4^+^-purified cells and then activated these and sorted again for T_regs _expressing surface LRRC32. Isolated LRRC32^+ ^or LRRC32^- ^T_regs _were then used in mixed lymphocyte response assays to assess the relative suppressive capabilities of each isolated population of T_regs_. Our results confirmed that naturally-occurring freshly-isolated LRRC32^+ ^T_regs _are more suppressive than LRRC32^- ^T_regs _(Figure 5d), exhibiting significant increases in suppression at T_reg_:T_eff _ratios of 1:4, 1:8, and 1:16 (*p *= 0.0324, 0.0142, and 0.0430, respectively).

## Discussion

The isolation of naturally-occurring functional T_regs _will be a prerequisite for successful adoptive immunotherapy techniques in humans. Murine adoptive immunotherapy models have demonstrated proof of concept that isolated T_regs _can be used as therapy for several autoimmune disorders [29-31]. The isolation of functional T_regs _in humans, however, is problematic, in part due to a paucity of specific surface markers. In this report, we demonstrate direct evidence of the surface expression of LRRC32 on freshly-isolated, naturally-occurring, and non-expanded CD4+ CD25^hi ^human T_regs _following TCR activation. We furthermore have characterized LRRC32 processing and demonstrate that sorted subsets of freshly isolated T_regs _bearing this marker appear more suppressive than subsets lacking this marker.

Previous studies have shown that constitutive over-expression of *Lrrc32 *in CD25^- ^T_effs _can lead to *Foxp3 *upregulation and that these cells subsequently acquire a suppressive phenotype [8,25]. Similarly, overexpression of *Foxp3 *has been shown to result in increased mRNA levels of *Lrrc32*, suggesting positive feedback between FoxP3 and LRRC32 [8,25]. Although other groups have shown that surface LRRC32 is highly elevated in expanded activated T_regs _compared to CD25^- ^T_effs_, we demonstrate here that low levels of intracellular LRRC32 are detectable in naturally-occurring freshly-isolated unstimulated T_regs _(Figure 1d) [8,25].

Previous studies utilizing an antibody generated against amino acids 296-308 of LRRC32 failed to detect LRRC32 on transfected Jurkat cells or on native CD4^+^CD25^hi^T_regs _[25]. However, as shown here, a commercially available anti-LRRC32 monoclonal antibody does recognize surface LRRC32 on transfected HEKs and naturally-occuring freshly-derived T_regs _that have undergone stimulation. Furthermore, it detects low intracellular expression of LRRC32 in naturally-occurring freshly-derived T_regs_. Failure to detect surface LRRC32 by the antibody raised against peptide 296-308 may be due to competitive occupation by a ligand as this region of LRRC32 corresponds to a loop and has been hypothesized to correspond to a ligand binding site [25]. One proposed ligand that could occupy this site may be LAP, as recently published work has demonstrated an interaction between LAP and LRRC32 [11,13,32]. If residues 296-308 of LRRC32 act as a binding site for LAP, occupation of this site may account for the failure of previous LRRC32-specific antibodies to recognize surface LRRC32 expression.

To determine if LRRC32 is sequestered in T_regs_, we examined the intracellular expression of LRRC32 in naturally-occurring freshly-isolated unstimulated T_regs _by flow cytometry. We show that unstimulated T_regs _contain low levels of intracellular LRRC32 protein. However, coupled with our RT-PCR studies showing high levels of *Lrrc32 *mRNA in unstimulated T_regs _relative to T_effs_, these data suggest that post-transcriptional mechanisms may play a role in controlling LRRC32 production and expression in non-activated T_regs_. Such post-transcriptional controls may be diminished upon stimulation via TCR/CD28 signaling, as indeed, upon stimulation, evidence of increased intracellular LRRC32 protein was evident in T_regs _as assessed by flow cytometry (Figure 1d).

In addition, our signal peptide deletion construct studies reveal that the putative signal peptide in LRRC32 is critical for the cell surface expression of LRRC32, consistent with other reports that signal peptides are necessary for surface protein expression [33]. Our data showing that LRRC32ΔSP is transcribed (Figure 4c) but not detected intracellularly (Figure 4b) suggest that LRRC32ΔSP is rapidly broken down in the cytosol or is not translated at detectable levels following transcription. However, mechanisms for the rapid degradation of misfolded proteins exist to maintain cell viability, and as such, cytosolic LRRC32ΔSP, unable to enter the endoplasmic reticulum owing to the lack of a signal peptide, may be translated but rapidly degraded afterwards by processes such as ubiquitination [34].

Finally, using freshly isolated, non-expanded CD4^+^CD25^hi^LRRC32^+ ^T_regs_, we show that such cells expressing surface LRRC32 appear to be functionally more suppressive than CD4^+^CD25^hi^LRRC32^- ^T_regs_. Previous reports have shown that upon activation of T_effs_, surface CD62L is usually decreased [35-37]. However, T_regs _normally maintain CD62L expression and functional phenotype [38]. Furthermore, previous reports have shown that CD62L^+^CD4^+^CD25^hi ^T_regs _are more suppressive than their CD62L^- ^counterparts [39,40].We show here that expression of surface CD62L appears to decrease significantly on LRRC32^+ ^T_regs _compared to LRRC32^- ^T_reg _populations.

Differences in CD62L processing may be responsible for the observed difference in CD62L expression between LRRC32^+ ^and LRRC32^- ^activated T_regs_. It has been shown that 90% of CD62L is rapidly cleaved from the surface within 4 hours of T cell activation prior to increasing over the next 48 hours, due to enhanced message stability, before ultimately decreasing due to downregulation of gene transcription [41]. Furthermore, it has been reported that CD62L is rapidly shed in T cells, including T_regs_, after activation [42,43]. In accordance, our data show that unstimulated LRRC32^- ^CD4^+^CD25^hi^FoxP3^+ ^T_regs _expressed more surface CD62L than stimulated LRRC32^+ ^or LRRC32^- ^CD4^+^CD25^hi^FoxP3^+ ^T_regs_. However, upon activation, decreases in surface CD62L expression of LRRC32^+ ^versus LRRC32^- ^cells were noted, suggesting that LRRC32^+ ^cells are more activated compared to LRRC32^- ^T_regs_. Given that an overnight stimulation is sufficient to induce LRRC32 expression on the cell surface of T_regs_, we chose this as our timepoint for phenotypic analysis. However, altering the time course of stimulation may also alter surface T_reg _marker expression.

Activated T_regs _that express LRRC32 may also represent a distinct population of more highly activatable T_regs _compared to LRRC32^- ^T_regs _[41]. Indeed, our phenotypic studies using LRRC32^+ ^and LRRC32^- ^subsets of T_regs _in the context of CD62L expression would appear to support the interpretation that LRRC32^+ ^T_regs_, relative to LRRC32^- ^T_regs_, are more prone to activation, as shown by increased cleavage of surface CD62L, and that this more highly activated state may translate into increased suppressive activity. Notably, although only a fraction of T_regs _expressed LRRC32 upon activation overnight, these cells appeared to be more functionally suppressive than their LRRC32^- ^counterparts.

Most natural FoxP3^+ ^adult T_regs _are CD45RO^+^, and the expression of CD45RO is typically a marker of T cell activation [44,45]. As LRRC32^+ ^T_regs _appear to be more suppressive than LRRC32^- ^T_regs_, it is possible that lower expression of CD45RO on LRRC32^+ ^T_regs _relative to LRRC32^- ^T_regs _may be due to increased auto-suppressive activity by LRRC32^+ ^T_regs _compared to LRRC32^- ^T_regs_. As noted above, stimulated T_regs _demonstrated expected increases in the surface expression of GITR, CD69, and CTLA4. GITR, or glucocorticoid-induced tumor necrosis factor receptor, was originally shown to be highly expressed on unactivated T_regs _but relative to T_effs_, and its expression was increased upon cell activation [46-48]. It appears that GITR is a co-stimulatory molecule, and although it is preferentially expressed on CD25^hi ^cells, it is also expressed at lower levels on T_effs_, and upon activation, T_effs _can also upregulate GITR [48,49]. Hence, the use of GITR as a specific marker for T_regs _appears to be limited. CD69 has been described in the context of a CD69^+^CD4^+^CD25^- ^T_reg _subset that does not express Foxp3 but does express surface-bound TGF-β1 in an ERK-dependent manner [50]. Normally, CD69 is upregulated upon T cell activation, and thus expected on our T_regs _[50-52]. Since LRRC32 also binds LAP, thereby helping to concentrate TGF-β1 at the cell surface, it is interesting to speculate whether CD69 in T_regs _may play a role in helping to upregulate TGF-β1 surface expression in the context of LRRC32 when the latter is available. Although our data did not find any significant differences in the CD69 expression in stimulated LRRC32^+ ^and LRRC32^- ^T_reg _subsets (Figure 5c), it is possible that part of the reason for the observed difference in suppressive activity between the LRRC32^+ ^and LRRC^- ^T_reg _subsets may be in part due to synergy between CD69 and LRRC32 via increased surface expression of TGF-β1. CTLA4, or cytotoxic T lymphocyte antigen-4, can inhibit T_eff _activation via 1) binding B7.1 and B7.2, thereby depriving CD28 on T_effs _of the ability to bind these co-stimulatory ligands, 2) inhibiting IL-2 transcription and progression of cells through the cell cycle via inhibition of cyclin D3, cdk4, and cdk6 production, and 3) decreasing the amount of time the TCR is engaged [53-57]. As we did not see any significant differences in CTLA4 expression in the LRRC32^+ ^and LRRC32^- ^T_reg _subsets, we do not have data to suggest that differences in CTLA4 expression might have contributed to the observed differences in suppressive function in the LRRC32^+ ^and LRRC32^- ^T_reg _subsets. Clearly, the results in this set of experiments raise many more interesting questions and suggest that the role of LRRC32 in the context of these other cell activation markers is complex.

Previous studies examining LRRC32 and T cell regulation have utilized T_effs _transfected with constructs containing either wildtype *Lrrc32 *or *Lrrc32 *lacking leucine rich repeat regions, the signal peptide, the cytoplasmic domain, or *Lrrc32 *with a mutated cytoplasmic residue postulated to be part of a PDZ domain and thus thought to bind an intracellular protein [8,25]. These experiments were performed to address how LRRC32 may be processed and ultimately function in T_reg _cells. PDZ domain mutation studies have suggested that the intracellular portion of LRRC32 is critical for surface expression, and other studies examined the LRRC32 deletion mutants in the context of downstream effector molecules such as FoxP3 [8,25]. These studies concluded that because FoxP3 expression was markedly decreased upon deletion of the leucine rich regions or the signal peptide, these regions were critical for LRRC32 function [8,25]. However, these studies never demonstrated actual cleavage of the putative signal peptide [8]. Here, we demonstrate via immunoprecipitation and confocal studies that LRRC32 encodes a signal peptide that is cleaved, and upon cleavage, allows mature LRRC32 to reach the cell surface.

It is likely that the LRRC32 signal peptide is cleaved from the newly translocated preprotein by type I eukaryotic endoplasmic reticulum signal peptidase, based upon the amino acid sequence of LRRC32 [58]. The initial amino acid sequence of LRRC32 incorporating a charged N-terminal domain followed by a hydrophobic domain is consistent with published reports of the consensus motif for eukaryotic type I endoplasmic reticulum signal peptidase [58,59]. Furthermore, the sequence G-L-A at positions 15 though 17 of the preprotein is consistent with the -3, -1 rule, stating that residues at the -3 and -1 positions, relative to the cleavage site, must be neutral and have small side chains [58].

## Conclusions

In summary, we have demonstrated a cleaved signal peptide site in LRRC32 is necessary for surface localization of native LRRC32 following activation of naturally-occurring freshly-isolated regulatory T cells. We show that LRRC32^+ ^CD4^+^CD25^hi^FoxP3^+ ^T_regs _express lower levels of surface CD62L compared to LRRC32^- ^CD4^+^CD25^hi^FoxP3^+ ^T_regs_, suggesting that LRRC32 expression may alter surface expression of other activation markers of T cells such as CD62L. Finally, functional data demonstrate that LRRC32^+ ^T_regs _appear more suppressive compared to LRRC32^- ^T_regs_, suggesting that LRRC32 surface expression may be useful as a marker that selects for more potent T_reg _populations, although our data suggest that the functional difference in suppression between these two populations is not markedly robust. Hence, LRRC32 selection may be most useful when used in combination with other T_reg _markers [60].

## Authors' contributions

DVC, AKS, ABY, JVM, JO, HS, and EG performed experiments represented in this manuscript. DB provided statistical support. KDC, HS, and TSM provided valuable designed the study. DVC, TSM, and KDC drafted the manuscript. All authors read and approved the final manuscript.
